# Ninety years of change on a low wooded island, Great Barrier Reef

**DOI:** 10.1098/rsos.181314

**Published:** 2019-06-19

**Authors:** S. M. Hamylton, R. McLean, M. Lowe, F. A. F. Adnan

**Affiliations:** 1 School of Earth, Atmospheric and Life Sciences, University of Wollongong, Wollongong, New South Wales 2522, Australia; 2 School of Physical, Environmental and Mathematical Sciences, The University of New South Wales, Australian Defence Force Academy, PO Box 7916, Canberra BC, Australian Capital Territory 2610, Australia

**Keywords:** drone, sand cay, rampart, mangrove, coral reef

## Abstract

We assess 90 years of change on a Low Wooded Island (Low Isles, Great Barrier Reef), employing drones and topographic profiling to accurately survey ramparts, mangroves, the reef flat and the sand cay. A comparison with maps from the 1928–1929 Great Barrier Reef Expedition revealed the redistribution of an outer rampart and inward movement of shingle ridges. Remarkable lateral expansion of the mangrove woodland some 400 m has occurred as carbonate sand deposition has increased reef flat elevation, obscuring coral microatolls. The sand cay has stayed relatively constant in size, moving approximately 44 m in a northeasterly direction and rotating slightly. We conclude that the existing configuration of landforms probably represents an equilibrium with local biophysical conditions, including sea level, wave dynamics, vegetation growth, storms and cyclones. The variable nature of ramparts and the presence of a trough that prevents the continuous spread of mangroves across a uniformly flat colonization surface precludes the interpretation of landform changes with respect to a geomorphic evolutionary sequence. Moreover, longer-term implications of environmental change for these landforms can only be evaluated once the specific nature of the local carbonate budget, including the relative contribution of corals, foraminifera and *Halimeda* has been elucidated.

## 1. Introduction

No coral island in the world has been so intensively studied over so long a time as Low Isles, the southernmost low wooded island of the Great Barrier Reef [1].

First described by Cook in 1770, with subsequent descriptions by King in 1819 and MacGillivray in 1848 (for a review, see [2]), Low Isles came to prominence as a base for investigations into biological problems on coral reefs when it was used as the headquarters for the 1928–1929 Great Barrier Reef Expedition. This was run jointly by the Great Barrier Reef Committee and the Royal Society of London [3]. A second expedition to the northern Great Barrier Reef (GBR), launched by the Royal Society and several Queensland universities, spent a number of days on Low Isles in August 1973 [4].

Over the past century, the changing biophysical nature of Low Isles has been interpreted in terms of long-term environmental controls such as sea level, and disturbances such as tropical storms [1,5–7]. Changes in the extent of mangrove cover, reef flat, moat ecology and associated alterations in the form and position of shingle ramparts and the leeward sand cay have been linked to fundamental questions about the processes responsible for reef top fauna and flora, habitats and landforms. These include episodic changes to the Low Isles reef in the wake of cyclones in 1934 and 1950, and movements of outer shingle ridges across the eastern reef flat some 20–30 m toward inner ramparts, smothering live corals and invertebrates in the process [6,8]. More recent studies reflect a growing concern for the changing environmental conditions surrounding the reef, including the influence of agricultural activities along the adjacent Queensland coast on water quality [7,9], and long-term monitoring of coral mortality in response to the impacts of coral bleaching, cyclones and outbreaks of crown-of-thorns starfish [10].

The past 90 years have seen remarkable developments in mapping technology, such that the activities that constituted ‘mapping’ on coral reef environments in the early twentieth century are now completely unrecognizable from those employed today [11,12]. The collection of published maps that depict Low Isles is unparalleled as a reliable body of work spanning a 90-year record (table 1). This record illustrates the incremental technological advances that have increased the efficiency, precision and accuracy with which the distribution of features such as mangroves, corals and islands can be recorded for a given point in time. For example, the highly detailed and well-known map produced during the 1928–1929 expedition by Spender [13], who used field-based methods of plane table and theodolite triangulation survey with the first aerial photographs of the reef, took eight months to prepare. For the present study, an image mosaic of comparable detail over the same complete reef system was acquired in 3 days using an aerial drone. For a given level of accuracy, coral reef mapping has now become easier, cheaper and faster. We draw on these developments to update the record of observation and change at Low Isles to span the 90 years since the 1928–1929 expedition. Although it is not a ‘typical’ low wooded island in the sense that it lacks the prominent, elevated conglomerate ridges and emergent microatolls that provide evidence of a higher Holocene sea level and can be found on many low wooded islands, the changes observed here can profitably inform a broader interpretation of low wooded island evolutionary dynamics on the GBR. We therefore outline several interpretations of the geomorphic evolution of low wooded islands to provide a theoretical context within which our observed changes to reef top landforms can be discussed.

**Table 1.** Previous mapping studies of Low Isles.

mapping study	reference
1. Physiographical sketch map of ‘Low Islands’ made by EC Marchant in 1928 for the purpose of discussing island formation using a plane table during the 1928–1929 Great Barrier Reef Expedition	Steers [2, fig. 5]
2. Coloured map of Low Isles surveyed by M Spender, Mrs TA Stephenson and EC Marchant in 1929 initially at scale of 1 : 2400 and published in the *Geographical Journal* in 1930 at scale of 1 : 5000 as fold-out map	Spender [12, Plate 1, follows p. 272]
3. 1931 reprint of Spender *et al*. coloured map of Low Isles, scale 1 : 5000, first published in the *Geographical Journal* but ‘with some minor corrections’ and additions of ‘Interior of the mangrove swamp from photographs by RAAF’	Stephenson *et al*. [14, Plate 1, follows p. 112]
4. Key chart of Low Isles based on Spender *et al*. map (2 and 3 above) in sepia colour with numerals, letters and arrows to identify specific features	Stephenson *et al*. [14, Text-figure 2]
5. Sepia map of Low Isles based on the survey by MA Spender in 1929 with modifications by FW Moorhouse in 1934 and RW Fairbridge and C Teichert in 1945	Fairbridge & Teichert [5, map follows p. 74]
6. Map of Low Isles depicting the sand cay, outlines of the reef platform and perimeter of the mangrove woodland and park made ‘by tape and compass traverse’ techniques during a 5-day visit to the island in 1973	Stoddart *et al*. [1, fig. 1, p. 64)

### 1.1. Low wooded island evolution on the northern Great Barrier Reef

Low wooded islands comprise a collection of landforms that have formed on planar reef platforms of relatively high elevation including windward shingle ridges and islands, elevated conglomerate platforms, mangrove swamps and leeward sand cays [15]. While similar islands are found in Indonesia, Jamaica, Polynesia, the Tuamotus and Belize, Stoddart (1973) noted that the 34 low wooded islands along the northern Great Barrier Reef offer the greatest possibilities for interpreting their evolution because of their diversity and their complex assemblages of landforms [4].

The present gross geomorphic form of the Great Barrier Reef islands reflects both their history of growth and their localized environmental setting. Microatoll evidence of a smoothly falling Holocene sea level from five low wooded island sites visited during the 1973 expedition to the northern Great Barrier demonstrated that this region of northern Queensland experienced a higher sea level in the mid-Holocene [16] and that this had exerted a fundamental influence on the geomorphology of reef islands [17]. A model of the Holocene reef development and formation of Bewick island identifies a ‘reef platform process window’ in which a critical water depth over the reef platform enhanced sediment production and transport to form islands within a rapid timeframe some 5000–4000 years BP [18]. Chronostratigraphic evidence from Pipon Island further north, which is also influenced by inshore terrigenous sediments, supports this model [19].

Over shorter timescales, observed changes to reef top landforms, including expansion of lagoonal reef flat mangroves and movements of sand cays and shingle ridges, have given rise to questions about the manner in which the reef top form is controlled by contemporary environmental conditions. Two distinct perspectives regarding this topic arose from the Royal Society expedition led by Sir Maurice Yonge in 1928–1929.

It is well known that the sea reached its present level on the northern GBR by about 6000 years BP [20]. Spender [21] was of the view that this time window was long enough for various elements of the reef top landforms to establish a stable equilibrium about which their distributions alternate in small oscillations between growth and destruction. Having visited many low wooded islands, his field observations included a wide variety of geomorphic transitions, including the relics of sand-rock movements, conglomerates, and occasional dead or dying mangroves, and the limit to expansion posed by the mangrove swamp. Spender interpreted this variation of behaviours across sites to indicate that each individual low wooded island ‘has reached for the given form of the reef and weather conditions a comparatively stable and balanced finality’ [21, p. 290].

An alternative viewpoint held by Steers & Kemp [22] suggested that the current configuration of reef top landforms can be interpreted as representative of a successional phase in a broader evolutionary sequence of reef platform infill. The starting point of this proposed sequence began after a time of higher sea level, as evidenced by a prominent platform or ‘bassett edge’ on many islands, when the sea reached its current level and stabilized there. This was followed by rapid mangrove expansion in which individual patches of mangrove coalesce from the site of the initial colonization on the windward, eastern side of the platform to join up with the sand cay on the leeward, western side. The endpoint of the evolutionary sequence was therefore reached when the expanding mangrove filled the entire accommodation space on the reef flat. The degree of mangrove cover on the reef flat was dependent on the size of the reef platform and indicated the stage of development of the complex. Stoddart *et al.* [1] noted later that this has little explanatory value in comparing low wooded islands because of the variations in physical conditions between them, drawing attention to the importance of random storm events in the destruction and formation of ramparts, which he argued were highly influential to reef flat geomorphology.

The 45-year assessment of change at Low Isles did not favour either of these viewpoints on the interplay between environmental conditions and reef flat geomorphic landforms. Rather, it highlighted the complexity of the recent history of low wooded islands, the difficulty of proposing simple models of their evolution and the need for continued monitoring to elucidate processes and rates of change [1]. Of particular note, Stoddart *et al.* [20] suggested a positive feedback mechanism under which rapid geomorphic changes, including mangrove expansion across reef flats, are initiated by the deposition of carbonate sediments. These play an important role in elevating the intertidal level of reef flats and reducing energy conditions, leading to mangrove colonization. Once established, the mangrove roots further dissipate energy, leading to the accumulation of windward shingle ramparts that provide further protection for mangroves from storms. Upon initiation, this collective set of incremental growth processes can rapidly replace bare reef flats with a complex array of landforms associated with low wooded islands, as observed in the 90-year cartographic record. Viewed in this way, Stoddart argued later that both Steers' view of an evolutionary progression and Spender's view of a series of equilibrium states could be reconciled depending on the time perspective adopted. While the comparative surveys of Low Isles reveal changes such as the expansion of mangroves as being opportunistic, short-term responses to elevations in substrate topography and protection from shingle ramparts, in the longer term such a process will invariably extend to cover the reef top.

Since the last major update of geomorphic changes to Low Isles, an increased awareness of the impacts of global climate change has raised further questions about how reef islands respond to phenomena such as sea-level rise, coral bleaching and ocean acidification, both historically and in the future. Globally, there are widely perceived notions that reef islands are some of the most vulnerable coastal landforms that will probably erode or even disappear by the end of the century [23]. This is despite several regional, multi-decade studies tracking changes across multiple Pacific Ocean reef islands that refute these ideas [24,25]. Indeed, a recent synthesis suggested that the vegetated core of islands is not expected to undergo significant modification, although beaches and the marginal seaward zones of islands may remobilize (e.g. island migration and narrowing) over the next century [26].

How the effects of coral bleaching and ocean acidification will be felt on reef-associated landforms ultimately depends on the extent to which reef top morphology relies on carbonate sediments produced by the organisms that inhabit the reef environment (e.g. corals, calcified algae and molluscs). While the accretion of reef platforms and the geomorphic integrity of associated landforms are fundamentally reliant on the reef carbonate budget [26], the implications of climate change for the geomorphic development of reef flat landforms are less well known. This is largely because of differences in the timescales of reef ecosystem health and geomorphic landscape responses to environmental changes [25]. For example, extended periods of elevated sea surface temperatures may see an immediately observable, dramatic expression in terms of coral mortality, yet the time taken for this to propagate through an indirect (or ‘nonlinear’) geomorphic pathway to give rise to a corresponding expression as an island shoreline movement is much longer. This makes the interpretation of geomorphic behaviours as a linked response to environmental drivers difficult. This difficulty is compounded by intermediate processes such as sediment production and transport that mediate this relationship. Furthermore, the magnitude of geomorphic impacts in term of factors such as sediment supply is likely to be smaller than more dramatic reductions in live coral cover [27].

Thus, the uncertainty of linking current observations and anticipated future projections of environmental change to associated behaviour of reef landforms emphasizes the value of retrospective studies that can provide some evidence-based insights into what can be reasonably expected in the future [25]. This study of landform changes at Low Isles can therefore provide valuable contextual information on the current modes of reef top geomorphic behaviour (e.g. sand cay migration, terminal spit fluctuation, rampart movements and mangrove expansion), which typify the many similar island types, both nationally and globally, onto which the effects of coral bleaching and ocean acidification will be superimposed.

### 1.2. Site description

**Figure 1.**
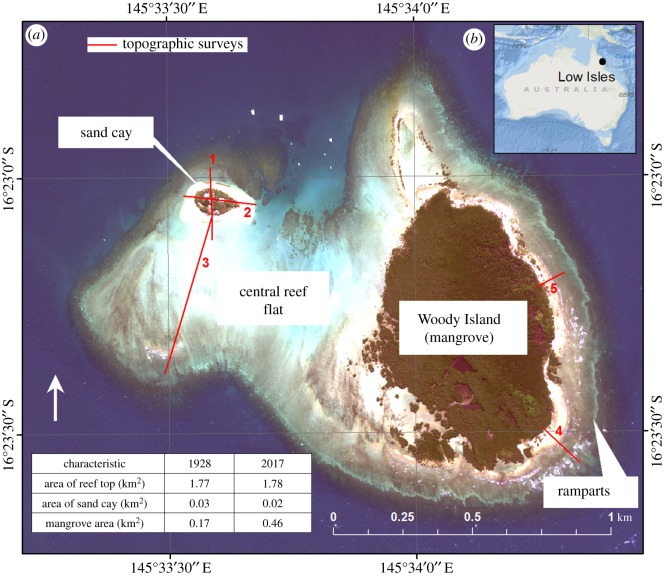
(*a*) Low Isles reef showing sand cay, central reef flat and Woody Island. The locations of transects surveyed in 2017 are identified in red, Worldview 2 satellite image, 24 July 2012. (*b*) Location of Low Isles in relation to the Australian continent. Inset table shows areas of reef top, sand cay and mangroves for 1928 and 2017.

Low Isles is situated at 16°23′ S, 145°34′ E on the inner shelf of the Great Barrier Reef (GBR), some 65 km north-northeast of Cairns and 15 km northeast of Port Douglas on the Queensland coast. The outer barrier of the GBR is 40 km to the east of Low Isles. A unique characteristic of the underlying reef platform shape of Low Isles is a southward-pointing indentation along the northern reef margin known as the ‘Anchorage’. This gives the reef its distinctive horseshoe-shaped formation, and supports a reef flat composed of ‘an uninhabitable mangrove swamp and an oval sand cay’ [3, p. 4] (figure 1).

The prevailing winds come from a southeasterly direction for the majority (85%) of the year, except during summer months (December to February) when the northwest monsoon dominates, characterized by heavy rainfall and occasional cyclones from the Coral Sea. A southeasterly swell transports sediments length-wise (i.e. in a northwesterly direction) across the lagoon [28].

Low Isles is the southernmost of a distinct type of GBR reef classified as a ‘low-wooded island’ [2] or ‘island-reef’ [29]. Based on the relative areal coverage of the windward shingle ridges, the leeward sand cay and the mangrove expansion across the reef flat, Low Isles can be classified as type 1, that is, a ‘*low wooded island with limited reef top mangroves and a separate sand cay*’ [17]. On the leeward, northwestern side of the reef there is a vegetated sand cay that is roughly oval in shape and covered to a large extent with a mixture of shrubs including *Tournefortia*, *Scaevola*, *Casuarina* and *Ipomoea* as well as a lighthouse and associated buildings. Approximately a quarter of the 1.78 km^2^ reef flat (0.46 km^2^) is covered by a mangrove forest, dominated by *Rhizophora* sp., and known as Woody Island, which lies some 600 m to the southeast of the sand cay. These are separated by a shallow trough or depression that lies across the northern central reef flat.

The majority of the carbonate production takes place around the periphery of the shallow outer reef slope, particularly on the windward side. Up on the reef flat, the main carbonate producers are the calcified macroalgae *Halimeda* and large benthic foraminifera that inhabit the algae and seagrass beds of subtidal ponds and around the reef crest [9].

Since 1928, several high energy events including storms and cyclones have been observed to generate and remobilize sediment around the reef flat, forming a series of inner and outer ramparts and associated shingle ridges comprising coarse coral fragments notably around the windward reef margin [5,8,30]. In places these ramparts intrude into the mangroves that comprise Woody Island, raising its elevation along the margins, for example, at ‘Green Ant Island’. The surface of this relict rampart intrusion in the east of Woody Island reaches approximately 4 m above sea level [5]. Stoddart *et al*., provide a review of previous studies of the biophysical character of Low Isles [1].

## 2. Methods

### 2.1. Methods overview

Fieldwork was undertaken at Low Isles from 8 to 12 August 2017. This combined aerial drone surveys with differential positioning accuracy to continue a 45-yearly update of observational changes to the ramparts, mangroves, central reef flat and sand cay using the best contemporary technology available. While several mapping exercises have recorded the distribution of reef flat features at Low Isles (see summary in table 1), this update to the cartographic record on the 90th anniversary of the Royal Society expedition to the Northern Great Barrier Reef (1928) represents one of the longest reliable global datasets on reef configuration.

Where possible, assessments of change went beyond two-dimensional comparisons of planimetric form to capture the complexity of three-dimensional volumetric adjustments through comparison of new topographic profiles with those measured during the 1973 expedition. Observed changes were interpreted with respect to broader questions of the manner in which geomorphic adjustments are taking place on the low wooded islands of the Great Barrier Reef.

### 2.2. Ramparts

The work of the 1928–1929 expedition was ‘greatly assisted by the very fine series of aerial photographs of Low Isles taken by flight 101 of the Royal Australian Air Force (RAAF) in September 1928’. A mosaic of some of these photographs is reproduced as Plate XXVII in [14, p. 19]. That mosaic is of the southeast corner of Low Isles and provides an excellent baseline of the historic configuration of features present along the eastern side of the reef top in 1928. This includes the reef flat, storm-deposited coral shingle ramparts, moats, ponds, littoral vegetation and mangrove swamp. In August 2017, a drone (DJI Phantom 4) aerial survey was flown over the same area at an altitude of 120 m to construct an image mosaic of the southeastern corner of Woody Island, including the ramparts, ponds, reef flat and mangrove swamp, for comparison with a mosaic of vertical aerial photographs of the same area taken in September 1928.

**Figure 2.**
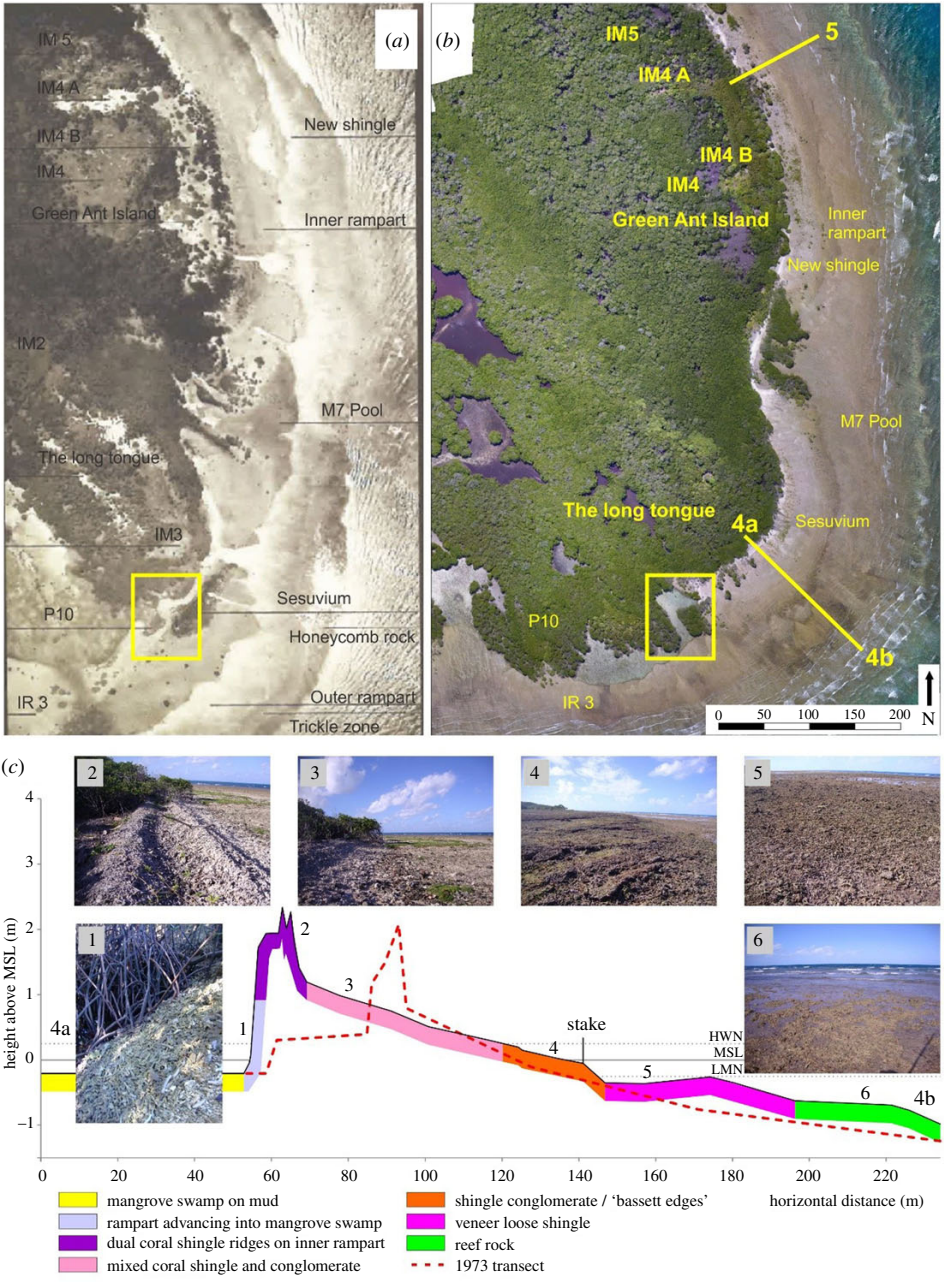
Aerial photographs taken of the southeastern corner of Woody Island in (*a*) 1928 (from [15], Plate XXVII) and (*b*) 2017, with a DJI Phantom 4 Pro drone. Specific geomorphic features are annotated, and the location of a prominent drainage feature that serves as a recognizable point of comparison between the 1928 and 2017 images is shown by the yellow boxes. (*c*) The elevation (m above/below MSL) and surface features of transect 4, showing corresponding elevations from the 1973 topographic survey (red dashed line) for comparison. The locations of the photographs are marked along the profile.

A topographic survey was undertaken using a Leica Sprinter automatic optical level across the ramparts in the southeastern section of the reef. This followed the same line as that surveyed in 1973 with a Kern automatic dumpy level during the Royal Society and universities of Queensland expedition to the northern Great Barrier Reef [4]. The two lines were co-located by means of a metal stake located on the reef flat to enable elevation and ground cover comparisons. This stake was emplaced prior to the 1973 survey, and its location is shown on the surveyed transect (figure 2*c*).

### 2.3. Mangroves

**Figure 3.**
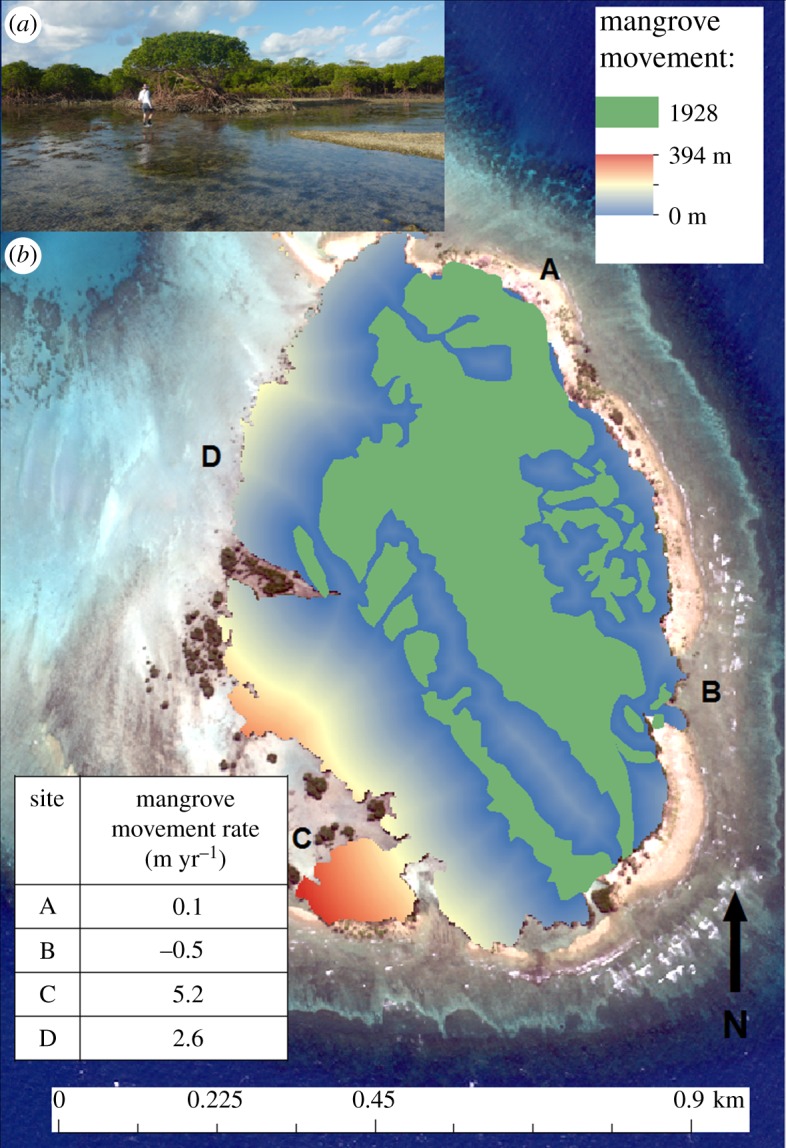
(*a*) Photograph of an isolated mangrove patch taken close to site C (foreground) with the laterally expanding southwestern mangrove front in the background and an advancing shingle rampart in mid-right that encloses a tidal moat; (*b*) A map showing the distance moved by the mangroves across the reef flat between 1928 (extent indicated by green shading) and 2017. Letters A to D indicate representative site locations around the mangrove periphery for which observed expansion rates are reported (inset table).

Changes to the mangrove woodland boundaries were mapped across the reef flat following a procedure applied for the same purpose at Diego Garcia atoll in the central Indian Ocean to derive a continuous estimate of shoreline change [31]. Briefly, the outer boundary of the Woody Island mangroves was digitized at a scale of 1 : 400 from both the geo-referenced 1928 map and the 2017 drone image. The Euclidean distance between mangrove boundaries on the different dates was computed using the spatial analyst proximity tools in ArcGIS 10.2. Lateral expansion rates were calculated for four individual sites at locations A to D as depicted in figure 3 around the mangrove boundary by dividing distance travelled by the time interval.

### 2.4. Central reef flat

A total of 6762 images were collected via drone at an altitude of 120 m across the entire Low Isles reef system over a period of the 3 days in August 2017 during which all fieldwork was undertaken. Approximately 4000 of these images were taken from the reef flat area between the sand cay and Woody Island at low tide, such that the reef flat features including live and dead coral, invertebrates and areas of sand, seagrass and algae were exposed. Images were processed using the photogrammetric software AgiSoft Photoscan [12].

Five topographic profiles were surveyed along ground transects, including the aforementioned rampart profile, the longest of which was a greater than 600 m traverse across the reef flat south-southwest of the cay (for locations, see figure 1). All surveys were undertaken using a Leica Sprinter automatic optical level and staff with GPS locations recorded at tripod positions. Field survey data were converted to Australian Height Datum (AHD) and local mean sea level (MSL) based on water level at the time of survey compared to the predicted tide level at Cairns at the same time.

### 2.5. Sand cay

An aerial drone survey of the sand cay was undertaken at low tide at an altitude of 60 m, and the resulting 140 images were stitched together using the aforementioned AgiSoft photogrammetry software to produce an orthomosaic of the cay (pixel resolution 2.38 cm, locational error 1.55 m). For further details on the drone survey and image processing, see [32]. As a basis for comparison, the 1928 Spender map was geo-referenced and the outlines of the beach, tidal sand spits, beachrock and vegetated sands were digitized in ArcGIS 10.4 at a scale of 1 : 100 m. For the geo-referencing exercise, the root mean square error, a commonly used metric for quantifying the locational error of maps [11], was found to be 0.23 m, which was small enough for the confident evaluation of shoreline changes.

## 3. Results

### 3.1. Ramparts

A detailed picture of the changes that have taken place along the eastern windward reef flat over the past 89 years can be gained through comparison of the 1928 aerial photomosaic with the 2017 drone image mosaic (figure 2*a*,*b*). These changes include further mangrove colonization to the south in the area of P10, and across the inner rampart at the southern tip of Woody Island, where the main body of the mangrove forest appears to have coalesced with the initial colonizing patches of *Rhizophora* evident in the 1928 photo (figure 2*a*).

Both Spender [29] and Stephenson *et al.* [14] described two ramparts, an inner and outer rampart, in this area in 1930 and 1931, respectively. In 2017 there was no outer rampart in the southeast or eastern segment of the reef flat from 200 m south of transect 4 to 300 m north of transect 5 (figure 1). Rather, there were three distinct zones across the eastern reef flat from low water neap (LWN) to high water neap (HWN). These were:

- bare surface of reef rock (formerly described as honeycomb rock, an organically cemented rock comprising sturdy branching corals cemented by crustose coralline algae with a flat upper surface [32]) and occasional live domal and branching corals;
- 50 m wide veneer of loose coral shingle over reef rock; and
- a 30 m zone of shingle conglomerate (see figure 2*c*, photos 6 and 5) and bassett edges (see figure 2*c*, photo 4).

The latter result from lithification of the leading edge of the shingle rampart through a cryptocrystalline high Mg calcite seawater cement, a process described in this area by Scoffin and McLean [32,33].

Some degraded remnants of the old indurated inner rampart were also present in 2017, located in the same position as it had been mapped in both 1973 and 1928, carpeted with *Sesuvium* and occasional dwarf mangroves (*Avicennia* and *Aegialitis*, see figure 2*c*, photos 2 and 3), similar to their antecedents in 1928–1929. A 1973 survey of one transect above LWN shows such a wall-like ridge 15 m wide and 2 m high separated from the mangroves by a 20 m wide coral rubble terrace 30–40 cm above the floor of the mangrove swamp, the terrace being the surface of a relic inner rampart. Our 2017 survey of this same transect indicates further northwesterly movement of the overlying shingle ridge some 40 m across the former inner rampart terrace and into the adjacent mangroves (figure 2*c*, red dashed line for 1973 ridge location). This mobile shingle ridge is part of the former outer rampart, as mapped in 1973, which has migrated inland to form dual coral shingle ridges over the inner rampart terrace as shown in the 1973 profile, which are now spilling into the mangrove swamp. The basal portion of this deposit (the old inner rampart) has not moved as shown on the two transects (figure 2*c*).

Discontinuous outcrops of inner rampart were backed by a ‘breastwork’ or bank of coral shingle well above HWN whose movement was first described by Moorhouse [30]. Further landward migration of these banks took place during the March 1934 cyclone [8] and probably during the 1950 cyclone [6].

Transects 4 and 5 comprise two or three minor ridges that suggest episodic development of the inner white coral rubble deposits indicating recent wash-over into the mangrove swamp (figure 2*c*, photos 1 and 2). Eastward migration of the mangrove swamp is constrained by the inner rampart, which rises directly from muddy substrate at a steep slope of some 60° to a crest around 2 m above the reef flat. While this distinct ridge and similar ridges along the inner margin of the eastern reef flat form a barrier to seaward migration of the *Rhizophora* woodland, further westward movement of the ridge is limited by the prop roots, stems and branches of the mangroves (figure 2*c*, photo 1).

### 3.2. Mangrove changes across the reef flat

Over the 90-year period, the area of reef flat covered by mangrove has expanded from 0.17 to 0.46 km^2^ (a 270% increase in area of reef flat covered). Moreover, internal expansion of the open glades in the mangrove swamp, described as ‘sinister places’ by Stephenson *et al*. [14, p. 39] such as at IM4 and IM5 to the west and north of Green Ant Island meant that there were completely covered by mangroves in 2017 (figure 2*b*). The peripheral boundary of the mangrove swamp (Woody Island) showed variability in the distance and rate of mangrove movements (figure 3). Mangroves along the southwestern aspect were associated with greatest rates of expansion of 5.2 m yr^−1^ over the 90-year assessment period at site C, which equated to a total distance of approximately 400 m over the 90-year period. This contrasted with net mangrove loss at a rate of −0.6 m yr^−1^ on the windward northeastern shoreline at site A.

### 3.3. Central reef flat

**Figure 4.**
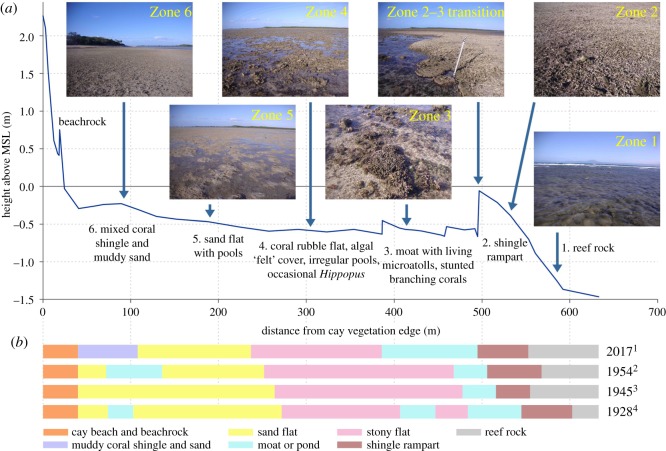
Transect surveyed from the sand cay across the western reef flat on 9–10 August 2017 (for location see transect 3, figure 1). (*a*) Profile of key habitats and surface sediment zones numbered 1–6, with photographs of each zone (see table 2 for descriptions). (*b*) Past zonation along same transect for 1928–1929, 1945 and 1958 based on maps and charts in: ^1^ (present survey); ^2^ ([5], figure 2); ^3^ ([4], Map facing p. 74); ^4^ ([16], Plate 1).

Between the mangrove swamp in the east and reef edge in the west is a broad reef flat subdivided by a low trough that drains northward into the main anchorage. In 2017 a transect was surveyed from the south side of the sand cay across the reef flat in a south-southwest direction (figure 1). This encompassed several different sedimentary environments and habitats that could be compared with features surveyed in earlier years (1928, 1945 and 1954) across the same traverse (figure 4). In 2017, six zones were recognized over a distance of greater than 600 m and elevation range of 1.24 m (see table 2 for detailed description).

**Table 2.** Description of habitat and sediment zones on the 2017 surveyed transect across the central reef flat of Low Isles. See figure 1 for location and figure 4 for profile details, images of zones and changes in surface features at time steps 1928, 1945, 1954 and 2017.

zone	width and elevation to MSL	description
Zone 1: Reef rock and live coral (formerly ‘Honeycomb rock’)	zone width 80 m elevation max = −0.70 m min = −1.47 m	Solid uneven reef flat pavement (‘honeycomb rock’) with encrusting coralline algal cover and discontinuous distribution of branching and domal corals emergent at low tide. Occasional dense thickets of *Halimeda*, brown filamentous algae, benthic foraminifera and clams. Zone of active carbonate production.
Zone 2: Shingle rampart (formerly ‘Outer rampart’)	zone width 58 m elevation max = −0.06 m min = −0.70 m	Rampart has typical asymmetrical topographic form comprising unconsolidated fragments of branching coral ‘sticks’ and Y-shaped clasts 10–20 cm in long axis and 1–2 cm diameter. Discontinuous outcrops of interior dipping, loosely imbricated bassett edges of similar sediments. Distinct rampart-moat edge is rhythmically cuspate in plan and convex in profile with variable steepness up to 50 cm high. Coral clasts are light-coloured and clean indicating active sediment movement.
Zone 3: Moat and discontinuous linear pools (formerly ‘Fungia moat’)	zone width 109 m elevation max = −0.46 m min = −0.67 m	Very shallow moat and discontinuous pools (<10 cm depth at low tide) with both dead and alive emergent clams (*Hippopus* and *Tridacna*) and several coral species including thin *Porites* microatolls up to 80 cm in diameter and patches of stunted *Millepora*, *Montipora* and *Pocillopora.* Microatoll and inter-coral surfaces typically covered with veneer of brown-grey mud and tufts of brown algae. Most of former ‘Fungia moat’ now occupied by advancing shingle rampart that forms the seaward barrier to Zone 3.
Zone 4: Coral rubble, stony flat (formerly ‘Thalamita flat’)	zone width 149 m elevation max = −0.56 m min = −0.63 m	Uneven coral rubble, stony surface with shallow pools (<5 cm deep at low tide) occupied by turf of brown algae, Holothurians, brittle stars. Components of coral rubble difficult to identify as entire area has covering of brown-grey mud. However, small massive stony corals (10–20 cm diameter) and clam shells dominate with branching coral fragments (as in Zone 2) uncommon.
Zone 5: Sandy mud flat (formerly ‘Sand flat’)	zone width 129 m elevation max = −0.31 m min = −0.56 m	Uneven surface of sandy mud and small shallow pools. Depressions and local mounds result from a number of burrows including shrimps, crabs, molluscs and worms. Average elevation of sandy mud surface is 0.5 m below MSL and 20 cm above Zones 3 and 4.
Zone 6: Mixed coral shingle and muddy sand (formerly ‘Sand flat’ or ‘Western moat’)	zone width 68 m elevation max = −0.23 m min = −0.31 m	Broad mound of muddy coral shingle and sand reaching an elevation 20–40 cm above Zones 4 and 5. Coral shingle fragments comprise both compact and branching forms having maximum diameters of up to 20 cm. All clasts covered with muddy sand. Likely source of coral clasts from western rather than southern reef.

The locations and surface characteristics of several parts of the reef flat mapped and named in the 1928–1929 expedition reports have changed over the 90-year period. These include the ‘Thalamita Flat’ in the west, the ‘Middle Moat’ around the southern and western reef rim moats, the ‘*Asterina* Spit’ around the shingle ramparts and the ‘Boulder Tract’. The ‘Mangrove Park’, a swampy area of scattered mangroves to the west of Woody Island, is now well to the west of where it was first mapped in 1928–1929 and ‘Thalamita Flat’ no longer consists of ‘sandy ground overlain by slabs and boulders of dead coral’ [14, p. 54]. Rather, it has been replaced by a stony flat in the north and a tidal moat in the south (see pink central portion of transect in figure 4*b* and discussion section for further detail). The northern half of the ‘Sand Flat’ is now occupied by a mixed coral shingle and muddy sand, while the ‘Boulder Tract’ in the extreme west of the reef flat is similar in both position and form, although additional reef blocks have been deposited by tropical storms.

### 3.4. Sand cay

**Figure 5.**
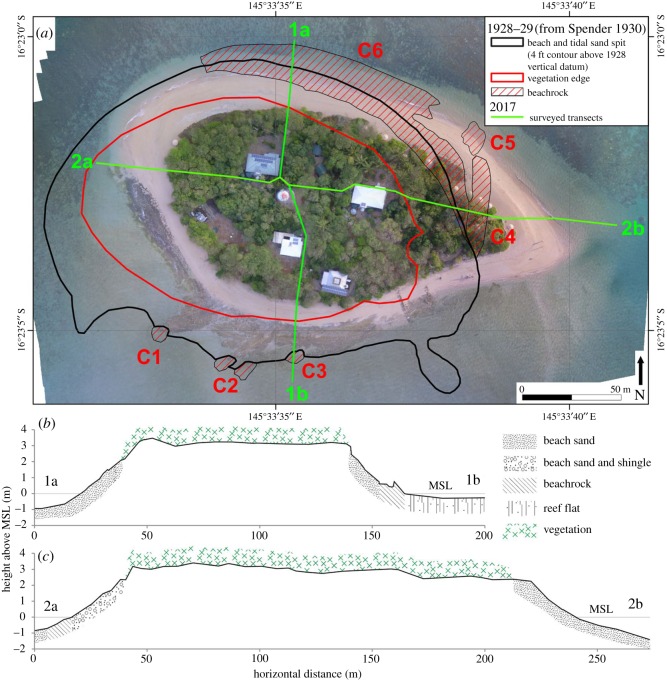
(*a*) Change at Low Isles sand cay since 1928–1929. The planimetric outline of the beach, beachrock and vegetation line as mapped in 1928–1929 are overlaid onto a mosaic of photographs acquired from a DJI Phantom 4 Pro drone survey in August 2017. (*b*,*c*) Elevation and surface topography of the sand cay along a north–south and west–east axis, respectively.

Based on a comparison of the location of the eastern vegetated tip, the sand cay has undergone a net movement of approximately 44 m over the past 89 years in a northeasterly direction, with a corresponding accumulation of sand covering previously exposed beachrock in the north, and erosion of sand exposing previously buried beachrock in the south. The overall vegetated area of the sand cay has reduced by a small amount (5 m^2^). The cay has also rotated because of erosion in the southwest and accretion in the east (figure 5).

Plan surveys of the vegetated part of the cay and surrounding beach were undertaken during the present survey (figure 5*b*,*c*). Elevations show that the beaches are slightly higher on the western and southern coastlines than the eastern and northern shores, by 80 cm and 1 m, respectively.

Surrounding the cay on the lower part of its slopes are various outcrops of ‘beachrock’ or ‘beach sandstone’; a rock formed by cementing of intertidal beach sand through the deposition of calcium carbonate between grains [34]. Six patches of beachrock mapped in 1928–1929 are shown in figure 5 (C1–C6). On the south side of the cay C1–C3 are clearly the protrusions of more expansive beachrock outcrops that were exposed during 2017, whereas the northern outcrops mapped in 1928 are now covered by the sandy beach. Further, Stephenson *et al*. [6] noted there was no trace of the eastern sector of beachrock (C5) in 1954 but the northern sector was still extensive, though the drone images in the present study indicate that this had been covered since 1952.

## 4. Discussion

In summary, the main changes observed over the 90-year period of assessment at Low Isles include the redistribution of outer ramparts on the eastern reef flat between transects 4 and 5 (figure 1), suggesting inward movement of former outer ramparts described in earlier studies [5,6,8]. The mangrove forest at Woody Island appears to have expanded in a westerly direction, with variable rates of lateral expansion (e.g. 5.2 m yr^−1^ in the southwest compared with 2.6 m yr^−1^ in the northwest). Changes to the character of the reef flat include deposition of sand and shingle in the north and boulders to the west, with the appearance of a tidal moat in the southwest. The leeward sand cay appears to have migrated approximately 44 m to the northeast and rotated slightly, while maintaining a constant area.

### 4.1. Rampart migration, reformation and shingle redistribution

The main differences between the early descriptions and aerial photos of the southeastern ramparts and the recent surveys include:

- redistribution of outer ramparts along the eastern sections of the reef flat,
- degradation of the inner rampart,
- inward movement of shingle ridges some 40 m across the reef flat (see 1973–2017 topographic comparison of Transect 4, figure 2*c*), and
- episodic movements of minor, recently deposited white coral rubble ridges related to storm and wash-over episodes and contained by mangrove roots and the elevation of the reef flat surface. Such ridges are also present to the northeast of Woody Island and along the southern shore of the central reef flat (figures 1 and 4).

The ramparts described by Stephenson *et al.* [14] and Spender [29] comprised low asymmetrical ridges of dead coral fragments heaped up to form a broad band of shingle some 60–80 m wide. The inner edge of the outer rampart was sharply defined as a steep escarpment about 0.6–1.2 m high that gradually sloped seaward, but in a landward direction occluded a tidal pool or moat 0.45 m deep at low tide containing living corals, molluscs and calcareous algae*.* Two examples of the outer rampart and pool labelled M7 Pool on figure 2*a* are illustrated in Stephenson *et al*. [14, Plate XI, fig. 1 and fig. 2]. At the time, the outer rampart contained fresh, white shingle indicating recent mobility of the deposit. This contrasted with the inner rampart of blackened (by a cover of cyanobacteria) coral shingle partly compacted by interstitial mud and sand and partly transformed into conglomerate, indicating stability of the rampart that in 1928 was about 180 m in from the reef edge.

It has been suggested that the dynamic nature of multiple ramparts across the reef flat reflects a cyclical process composed of successive periods of accumulation of debris [14,29]. This material will only be carried by waves a certain distance over the reef flat before their energy becomes dissipated and the debris is deposited. Higher energy events (e.g. storms and cyclones) will gradually cause the rampart to move inwards, until a new system develops outside the first. Over time, this may be followed by second, third and fourth ramparts each more or less parallel to the other a little farther to the exterior edge of the reef platform [5]. In 1945, Fairbridge & Teichert [5] described four such ramparts, the first and innermost being Green Ant Island, the second and third being the ‘inner rampart’ and ‘outer rampart’ of Stephenson *et al.* [14] and the fourth the outermost rampart formed since 1928. All four ramparts were concentrated within a 110 m wide zone along the eastern edge of Woody Island and set back 80–120 m in from the reef edge. An element of complexity is added to this proposed cycle, whereby no single rampart is necessarily forming consistently at a given point in time along its whole extent. Because the reef platform comprises multiple localized zones of varying degrees of exposure, as one rampart traverses around the reef, additional ramparts may begin to form outside it in exposed parts, while that same rampart is still accumulating in more protected areas. With this in mind, the ramparts mapped in the 1973 expedition were classified according to their colour (white versus grey versus black shingle) as a proxy for the sequence of ridges from seaward to landward. Rather than being a permanent feature of individual ridges, their colour can be used to identify mobile ramparts (white) from those recently stabilized (grey) and the older deposits [1, p. 64] as remobilization of ridge clasts during storms may result in the removal of the surficial grey or black cyanobacteria through inter-clast abrasion.

Our observations of the redistribution of outer ramparts, degradation of inner ramparts and inward movements of shingle ridges largely concur with those made by Frank & Jell [7], who suggest that tropical cyclones have primarily been responsible for transporting shingle inward to the margins of the mangroves. A superimposed series of eight maps depicting shingle ramparts digitized from aerial photographs along the eastern windward margin of the reef [7] suggests a highly dynamic system driven by the aforementioned processes, and indicates the presence of ramparts in 1991, which had disappeared by 2001 [12, fig. 4]. Wave uprush and backwash of seas that are reflected back from the steep and high inner coral rubble ridge (figure 2*c*) have probably elevated the hydrodynamic energy levels in this zone of the reef flat, preventing deposition and reformation of an outer rampart in this eastern area over the past two decades. Moreover, the formation of the ramparts observed in 1991 may have been related to Cyclone Ivor (March 1990) that passed over Port Douglas some 15 km to the southwest, which was the most recent cyclone to pass Low Isles. As this cyclone approached from the southeast, it could have produced waves of sufficient height and orientation to generate ramparts on the windward reef flat. Evidence of former ramparts in this area is widespread with one example being the extensive outcrops of bassett edges, the residual partially lithified foreset beds of previous shingle ridges [32], as shown in transect 4 (figure 2*c*, photo 4).

As also noted in the 45-year assessment of changes at Low Isles [1] it would appear that the formation and destruction of unconsolidated rampart features occurs in rapid enough geological terms to render them insignificant as indicators of historic sea levels, in contrast to the older, more strongly lithified conglomerate platforms observed at other low wooded islands. Examples to the north of Low Isles at Three Isles and Nymph Island date back to a time of high sea level in the mid-Holocene [15,20,35].

### 4.2. Mangrove swamp: rates and manner of expansion

Our data together with the 1973 survey [1] suggest that peripheral expansion of the core mangrove forest to the west has been incremental with areal increases from 16.8 ha in 1928–1929 to 36.5 ha in 1973 to 45.5 ha in 2017. These increases clearly contrast with Macnae's view that no significant change in mangrove area had taken place between 1928–1929 and 1964 [36]. Our topographic survey data indicate that the Low Isles mangroves extend over a large vertical range from the level of high water downwards to the lowest ones at low tide (approximately +1.5 m above MSL to −0.5 m). The elevation range of wetlands is determined by the localized tidal range, and with a tidal range of 2.3 m, the Low Isles mangroves are classified globally as macrotidal wetlands. This range exceeds the generally narrow range within which mangroves persist [37]. Indeed, in ponded environments within the shingle ramparts in nearby Three Isles and Low Wooded Island, Stoddart [38] recorded *Ceriops* mangroves at 3.46 m and 4.37 m respectively above datum [39], a range that is quite exceptional when compared with mangroves that reach 2–3 m above datum on the adjacent mainland coast at Cairns [40].

The remarkable lateral expansion of the Woody Island mangroves some 400 m in a southwest direction across the reef flat has been possible through increases in the vertical elevation of the reef flat, itself driven by deposition of sand and shingle in the lower energy, protected leeward areas of the mangrove woodland. As the reef flat gradually accretes upward into a vertical range suitable for mangrove colonization, the patchy distribution of mangroves suggests that *Rhizophora* seedlings become established where conditions are conducive to settlement. Broadly, these include low to moderate levels of rainfall and associated salinity levels, tidal inundation and limited subsurface current flows over the reef flat [41]. Overall, this manner of reef flat colonization contrasts with the idea of an ‘advancing front’ expanding from the existing wooded area of reef flat, as is typical of horizontally prograding mangroves in deltaic environments [39].

A similar situation occurs in the extreme northeast of Low Isles, where Stephenson *et al.* [14, text fig. 2] identified and mapped a ‘lonely mangrove’ in 1928–1929 between the northeast Moat and *Porites* Pond. It was in this area that the 1934 cyclone had its greatest impact with the movement of the shingle rampart westward by 25–30 m covering the pond surface with a flattened shingle deposit. Prior to the cyclone, the moat had been rich in the coral *Montipora* and clam *Hippopus*, but after the cyclone these had been smothered by shingle from the east [8, pp. 38–39]. A decade later Fairbridge & Teichert [5, p. 275] noted that *Porites* Pond had been encroached upon, this time from the west, by shingle from the adjacent Tripneustes Spit. They also noted that ‘Recent *Rhizophora* seedlings are sparsely distributed over the northern part of *Porites* Pond’. Later, these seedlings were reported to have died [6, p. 309]. Seven *Avicennia* and 12 *Rhizophora* mangroves were counted during the 1973 expedition along the eastern and western sides of the pond respectively. By 2017 there was a continuous fringe of mangroves (primarily *Rhizophora*) comprising approximately 211 individuals visible from the drone imagery, extending all the way around the former *Porites* Pond and including individual plants up to 3.0 m high. This recent and rapid expansion has been accompanied by infilling of the pond such that most of the former *Porites* microatolls are now dead and covered with sediment. Interpreting the broader history of mangroves across low wooded islands on the northern GBR, Stoddart [38] notes the occurrence of older fossil microatolls beneath more extensive mangrove swamps (e.g. at Leggatt, Houghton and Hampton Islands), suggesting that these represent a high-standing reef top some 5000–6000 years ago. In contrast, younger microatolls are associated with more rapidly expanding mangrove forests found on more modern ‘catch-up’ reefs. We suggest that the increasing cover of mangroves across the *Porites* Pond represents a contemporary example of this latter sort.

While Fairbridge & Teichert [5, p. 85] speculated ‘that it would only be a question of time until most of the reef flat would be covered with dense mangrove growth’, this possibility is unlikely to take place over the next few decades. The temporal series of aerial images and maps accurately illustrate the mangrove periphery and associated areas in 1928 (0.16 km^2^), 1973 (0.36 km^2^), 2012 (0.45 km^2^) and 2017 (0.46 km^2^), thereby providing an opportunity to calculate average rates of mangrove expansion over the intervening time periods. Average annual expansion rates appear to have fallen from 4.4 m^2^ yr^−1^ in the period 1928–1973 to 2.18 m^2^ yr^−1^ in the period 1973–2017, the latter figure being corroborated by the shorter 5-year window of expansion captured between the 2012 satellite image and the 2017 drone image, during which the average yearly expansion rate was 2 m^2^ yr^−1^. Such temporal variability in the rate of mangrove expansion highlights the dynamic nature of the physical processes driving expansion, drawing attention to the absence of a simplified set of consistent forces driving the evolution of the low wooded islands towards a common endpoint, as proposed by Steers & Kemp [22].

At Low Isles, expansion of the mangrove front in a northwesterly direction across the reef flat is largely precluded by the deep north to south trough that separates the Woody Island mangroves from the western reef flat. Possible future changes in environmental boundary conditions including sea-level rise and a longer term reduction in the source of calcium carbonate sediments arising from coral mortality due to bleaching may compound this situation. The utility of the relative extent of mangrove coverage on the reef top as a basis for comparing the evolutionary development of a range of low wooded islands [2] is therefore limited.

### 4.3. Changes to the central reef flat character

While Steers [2, p. 15] described the reef flat between the sand cay and the mangroves as ‘simply the flat sandy top of a reef’, Stephenson *et al*. [14, p. 25] noted that the central reef flat is ‘by no means uniform in structure or appearance’. The latter was also the case in 2017, when the general pattern of diverse habitats and landforms was clearly recognizable in the drone images and in the field.

In 1928–1929 the two largest areas of the reef flat were Thalamita Flat and Sand Flat. Of the former, Stephenson *et al*. [14, p. 25] noted that its ‘characteristic feature is the presence of numerous slabs and boulders of dead coral rock with sand and pools between them’. Similarly, Stephenson *et al.* [6, p. 281] found Thalamita or Stony Flat ‘characterised by flat slabs of dead coral (*Acropora*), interspersed with horseshoe clams (*Hippopus*) and occasional coral boulders’. We now know that most of these ‘slabs’ of dead coral or coral rock are fossil microatolls first recognized during the 1973 expedition when Scoffin & Stoddart [34] noted that in broad shallow pools of the central part of the reef flat ‘large thin microatolls grow and commonly coalesce to fill the pools with a flat coral pavement’.

The second largest area of the reef flat was described by Stephenson *et al*. [14, p. 25] as the Sand Flat: ‘an area of sand with shallow pools’ that extended in an arc from the sand cay some 230 m to the south. In 2017, we identified two quite distinct zones in this area. One of these adjoined the former Stony Flat and comprised a muddy sand surface with pools (see figure 4*a*, photo 5). Further north, the other had a much more variable substrate including dead branching coral fragments 5–10 cm in long dimension and coarse sand both with a ‘felty’ and muddy algal cover (see figure 4*a*, photo 6).

Between the Thalamita Flat and the outer reef slope on the southern rim of Low Isles, three parallel zones were identified, mapped and described in 1928–1929: Fungia Moat, Outer Rampart and Honeycomb Rock. All three zones were identified in 1933 [30], 1945 [5] and 1954 [6]. They were also recognizable in 2017, but importantly their location has not been stable but has shifted northward (figure 4*b*) particularly through shingle rampart erosion and wash-over deposition. For instance, the leading edge of the advancing shingle rampart and its inclined slope has almost completely covered the former Fungia Moat to within a few metres of its earlier northern boundary. As a result the 30–49 cm high leading edge has been of sufficient height to provide a barrier to maintain a linear pond that has enabled coral growth of thin *Porites* microatolls and stunted branching corals *Montipora* and *Millepora* (as shown on figure 4*a*, photos 2 and 3). Future maintenance of this moat coral and rampart morphology is unlikely as the increasing surface elevation of the potentially receiving surface of the former Thalamita Flat is above the level of surface ponding and continued coral growth (figure 4*a*). The erosion of shingle ramparts and dispersive influence of wash-over along the southern portion of the central reef flat may reflect the extended period of relatively low energy ‘baseline’ conditions in which hydrodynamic regimes are largely governed by the southeast trade winds, it being 28 years since Tropical Cyclone Ivor passed through the region.

### 4.4. Sand cay size and position

Comparison of the cay as mapped by Spender and described by Stephenson *et al*. [14] during the 1928–1929 expedition with the drone image mosaic generated by our study indicates a small reduction in cay area (5 m^2^, or less than 1% of the original cay size). Based on the edge of the vegetation line, the cay has also migrated some 44 m to the northeast. Such changes are ephemeral as the size and position of the sandy and rocky area around the cay change seasonally and periodically in line with prevailing winds, cyclones and exposure to incident waves [42]. Indeed, Fairbridge & Teichert [5] report a cyclone that destroyed some of the trees and buildings on the sand cay in 1934, noting that the physical position was restored to approximately the original state by 1936. While low-lying reef islands are widely perceived to erode in response to measured and future sea-level rise, a dynamic range of island changes has been recorded in global studies across the Atlantic, Indian and Pacific oceans over the past few decades [24,43,44].

Some ‘characteristic’ sand cay behaviours include alongshore drift, extension of the ends of elongate islands [45] and migration in the position of islands on reef platforms, a comparable example being the 35 m movement of the leeward sand cay Three Isles, a low wooded island 150 km to the north [46]. Circulatory movements of sediments driven by seasonal winds around island peripheries have been observed at Poruma Island in the Torres Strait [47], which commonly give rise to oscillatory motions in the terminal spits of oblongate cays, as have also been observed at several islands further south on the GBR. Variability in the movement of peripheral sediments (i.e. erosion versus accretion) around the shorelines may arise from the stability imparted by beachrock ‘which acts as a natural bulwark’ [5, p. 88] or the root structures of vegetation such as the coconut trees [48]. Moreover, the relatively constant areal footprints of both the vegetated cay and the ‘toe of beach perimeter’ over the past 90 years indicates the possible presence of an equilibrium in the cay's position and size. In short, we agree with Spender's conclusion that the Low Isles cay is maintained by a nicely balanced equilibrium of forces of the prevailing winds and waves, with episodic storms and tropical cyclones.

### 4.5. Carbonate dynamics at Low Isles reef

The longer-term outlook for Low Isles will ultimately depend on the interplay between the carbonate budget and biophysical processes including wave dynamics, sea level, vegetation growth and episodic storms and cyclones. Frank [28] characterizes the most recent stage of Low Isles reef development (*ca* 2000–0 years BP) as having extremely low rates of carbonate production and a dominance of destructive reef processes. Recent mass coral bleaching in the northern and central Great Barrier Reef [49], which has driven temporary collapse in reef carbonate budgets and coral growth elsewhere [50] may compound this situation. However, we observed that both the upper reef flat and forereef slope continue to support a diverse live coral community, particularly on the windward side as indicated by underwater video camera footage (electronic supplementary material, figure S1) and the presence of domed *Porites* bommies among the reef rock along the outer reef flat. Furthermore, the relative contributions of other carbonate producers at Low Isles, including foraminifera [9] and *Halimeda* [51], which can represent a significant portion of the carbonate budget, may determine the extent to which these landforms are impacted by future environmental changes.

## 5. Conclusion

In summary, the 90-year assessment of change revealed the following notable changes to the Low Isles reef flat:

1. Episodic movements of mobile shingle across the reef flat, including the redistribution of an outer rampart and inward movement of shingle ridges.
2. The mangrove forest occupying the windward side of the reef flat known as ‘Woody Island’ expanded laterally some 400 m as the reef flat has become elevated through carbonate sand deposition, with notable mangrove colonization around the periphery of the area known as *Porites* Pond.
3. On the central reef flat surface, changes to the surface composition included disappearance, by sediment coverage, of many coral microatolls and the introduction of muds and shingle to the northern sand flat that abuts the southern shoreline of the sand cay.
4. The sand cay itself has stayed relatively constant in size, but moved approximately 44 m in a northeasterly direction and rotated slightly.

It is clear that sufficient time has elapsed since sea level reached its present elevation for the existing configuration of these landforms to have achieved an equilibrium with the physical environmental processes that shape them (e.g. wave and sediment dynamics) [28]. To this end, some of the changes observed in the present 90-year assessment represent ‘typical’ geomorphic behaviour of coral reef landforms observed elsewhere. These include movements of shingle ridges and spits in response to high energy events [52] and island migration and rotation across reef platforms [24]. Such behaviours are dynamic fluctuations in response to local environmental conditions, rather than unidirectional changes occurring within a broader evolutionary sequence as posited by Steers & Kemp [22], to which the entire collection of low wooded islands on the Great Barrier Reef adhere. Although the reef top landforms at Low Isles, as with the rest of the low wooded islands, have invariably developed from antecedent Pleistocene reef platforms, we interpret their current degree of complexity and landform configuration, and the changes that can be elucidated from comparison to their historical configurations, to be an expression of local geomorphic and environmental conditions.

## Supplementary Material

Supplementary material 1

## Permission to carry out fieldwork

Fieldwork was carried out under Great Barrier Reef Marine Park Authority and Queensland Parks and Wildlife Service Permit G17/39268.1.

## Data accessibility

The primary data from this study, including the aerial drone mosaic and five topographic profiles, are available in the Dryad Digital Repository at: https://doi.org/10.5061/dryad.2t8n4sd [53].
