# Supplementary material for: Ninety years of change on a low wooded island, Great Barrier Reef
Source: R Soc Open Sci. 2019 Jun 19;6(6):181314. doi: 10.1098/rsos.181314 (PMC6599766; doi:10.1098/rsos.181314)

**Supplementary Material: Figure 1.** Coral cover observed using underwater video cameras* around the peripheral forereef of Low Isles (June 2017).

* coral communities were viewed from the boat by lowering an underwater drop camera into the water column at a series of site around the reef periphery to collect video footage of the reef platform. Individual drop camera surveys lasting acquired short (approximately 1 minute) snapshots of oblique underwater video footage over a wide range of reef environments (e.g., forereef slope, reef crest, shallow reef flat etc.), The geographical position of each footage sample was recorded with a Global Positioning System device (GPS). All drop camera video footage was subsequently viewed and percentage cover estimates of coral were generated for each survey point.


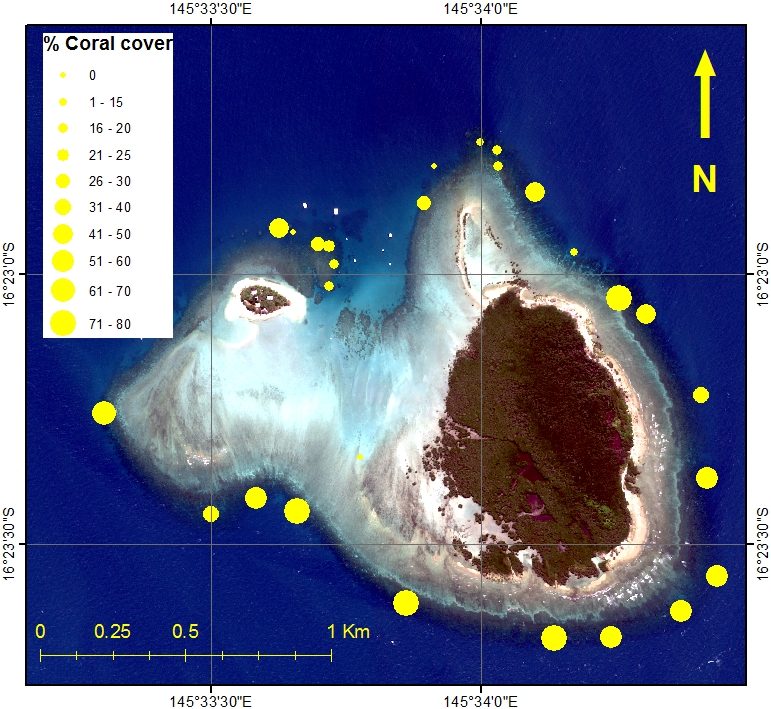

Supplement: Supplementary material 1 [file rsos181314supp1.docx]
